# Covid-19—Beyond virology: Potentials for maintaining mental health during lockdown

**DOI:** 10.1371/journal.pone.0236688

**Published:** 2020-08-04

**Authors:** Aisha J. L. Munk, Norina M. Schmidt, Nina Alexander, Katrina Henkel, Juergen Hennig

**Affiliations:** 1 Department of Differential and Biological Psychology, Faculty of Psychology and Sport Sciences, Justus-Liebig University Giessen, Giessen, Germany; 2 Department of Psychology, Faculty of Human Sciences, Medical School Hamburg, Hamburg, Germany; Qazvin University of Medical Sciences, ISLAMIC REPUBLIC OF IRAN

## Abstract

**Background:**

The current study aimed to assess prevalence of mental disorders during Covid-19 pandemic- and respective lockdown in Germany, and potential behaviors/states that can have protective functions on preventing severe mental problems. Assessing prevalence of mental disorders, as well as to find potential protective variables is very important in order to determine people’s psychological suffering. It provides the basis for teaching possible coping styles in order to prevent a major breakdown on mental health. Prevalence on mental disorders was expected to increase during the pandemic, especially depression, (general-/and health-) anxiety, panic attacks- and disorder, as well as obsessive-compulsive disorder. Additionally, potentially protective variables, such as resilience and coping, were included.

**Methods:**

N = 949 subjects completed an online-survey that asked for symptoms regarding depression, (health) anxiety, panic disorder, obsessive-compulsive disorder, and lock-down related behavior—starting 14 days after lockdown in Germany.

**Results:**

Prevalence of mental disorders in the current sample was much higher than usual prevalence of mental disorders, with 50.6% expressing at least one mental disorder. Resilience was associated with lower risks for any mental disorder (OR = 4.23, p < .0001, 95%CI = 3.21–5.57), as well as with any other measured mental illness (all ORs between = 2.82 for obsessive-compulsive disorder and OR = 41.44 for panic disorder, all p < .001). Similar results were obtained regarding coping (focus on positive).

**Conclusion:**

Results are highly relevant in order to provide a glance on what substantial influence the current pandemic- and lockdown situation has on mental health across the country, and possibly across the world. Possible ways in order to prevent deterioration and help coping with the current situation are being elaborated and discussed.

## Introduction

For what is known so far, severe acute respiratory syndrome corona virus 2 (SARS-CoV-2) has begun to spread in Germany since beginning of March 2020. It was first reported of in December 2019 in Wuhan, China [1] and has since then begun to expand across the globe. Whereas most infected people show mild symptoms (≈ 80%), some develop pneumonia (≈ 14%) and/or acute respiratory distress syndrome (ARDS; ≈ 6%, Robert-Koch-Institute, Germany). Eventually many of those severe cases need intensive care [2, 3]. As the virus spreads very fast, many people are being infected in a short time [4].

Because of a possible health-system breakdown after a sudden and substantial increase of patients needing intensive care/intubation, severe restrictions have been put up in most countries facing SARS-CoV-2. In Germany, schools/kindergartens/universities, restaurants, etc. have been closed, and many people are working from home: Public social life has basically stopped existing.

Therefore, providing a first overview of the most prevalent mental health problems at this emergency situation’s early stage will help establishing a targeted support-and treatment-system, and will further allow a prognosis regarding the expected disease-burden for affected societies.

One of the first studies concerning the corona-outbreak and mental health is from Gao et al. [5], reporting high prevalence of mental health problems in China in February 2020: 21.3% of the sample expressed psychological problems/anxiety. Since then, there is a growing body of literature regarding mental health during the COVID-19 outbreak, reporting psychological problems like depressive symptoms, anxiety and decreased quality of sleep in different populations [6–9].

However, most of the available literature only reflects results of mostly Asian countries [9], thus, leaving a need for representative studies also from European- and other areas across the globe–all of which are currently dealing with coronavirus-outbreaks. To our knowledge, no study presenting results of mental health problems with a larger sample in Germany during the Sars-Cov-2 outbreak- and lockdown has been published so far.

In order to assess the current situation's impact on mental health and to be able to respond as quickly as possible, we conducted an online survey on N = 949 participants. We assessed health anxiety/somatoform disorder (HA), general anxiety disorder (GAD), panic disorder (PAD), obsessive-compulsive-disorder (OCD), and depression (DEP). Furthermore, questions regarding life circumstances during the nation’s “lockdown” were measured, as we expected substantial influence of it. Generally, we expected current prevalence of assessed mental disorders to be higher than the usual one in Germany [10]. We considered coping and resilience as behaviors being protective against adverse effects on mental health. Furthermore, in order to reveal emotional and psychological changes throughout the development of the disease, the sample will be longitudinally followed. For what is known so far, severe acute respiratory syndrome corona virus 2 (SARS-CoV-2) has begun to spread in Germany since beginning of March 2020. It was first reported of in December 2019 in Wuhan, China [1] and has since then begun to expand across the globe. Whereas most infected people show mild symptoms (≈ 80%), some develop pneumonia (≈ 14%) and/or acute respiratory distress syndrome (ARDS; ≈ 6%, Robert-Koch-Institute, Germany). Eventually many of those severe cases need intensive care [2, 3]. As the virus spreads very fast, many people are being infected in a short time [4].

Because of a possible health-system breakdown after a sudden and substantial increase of patients needing intensive care/intubation, severe restrictions have been put up in most countries facing SARS-CoV-2. In Germany, schools/kindergartens/universities, restaurants, etc. have been closed, and many people are working from home: Public social life has basically stopped existing. Therefore, providing a first overview of the most prevalent mental health problems at this emergency situation’s early stage will help establishing a targeted support-and treatment-system, and will further allow a prognosis regarding the expected disease-burden for affected societies.

Next, we considered coping and resilience as behaviors being protective against adverse effects on mental health. Resilience refers to positive adaptation, or the ability to preserve or regain mental health, in the face of experiencing difficulties in life [11–14]. Coping is viewed as a response to perceived stress and defined as "constantly changing cognitive and behavioral efforts to manage specific external and/or internal demands that are appraised as taxing or exceeding the resources of the person" ([15], p.141). The way of perceiving and interpreting events and differences in dealing with those have been shown to have different impacts on mental health- an effect that can be found across all ages [12, 16–22]. Furthermore, in order to reveal emotional and psychological changes throughout the development of the disease, the sample will be longitudinally followed.

To allow the health care system to respond as quickly as possible, the current study provides an early overview of the most prevalent mental health problems, and of strategies that proofed to be protective regarding mental health problems. We expect

- Higher prevalence of all measured mental disorders in comparison to the usual numbers in Germany [10] (namely DEP, GAD, PAD, HA, and OCD), due to the ongoing threatening, unclear situation
- Associations of lockdown related behavior (social isolation, hoarding, worrying) with mental disorders
- More resilient people being less affected by the lockdown in regard to mental disorders
- Mental health outcomes being differentially associated with coping, especially focusing on positive sides of the situation.

## Materials and methods

### Design and participants

In order to assess the current situation's impact on mental health and to be able to respond as quickly as possible, we conducted an online survey on N = 949 participants. We assessed health anxiety/somatoform disorder (HA), general anxiety disorder (GAD), panic disorder (PAD), obsessive-compulsive-disorder (OCD), and depression (DEP). Furthermore, questions regarding life circumstances during the nation’s “lockdown” were measured, as we expected substantial influence of it. Generally, we expected current prevalence of assessed mental disorders to be higher than the usual one in Germany [10].

This cross-sectional data come from the first wave of a prospective online-survey conducted between March 27th and April 3rd, 2020. A link to the survey was posted on social media, and sent via university e-mail to members of Justus-Liebig University of Giessen, Germany (with the request to answer some questions regarding consequences of the Covid-19 pandemic). In total, 949 participants completed the survey. Subjects were asked if they agreed to participate in a follow-up study, and if agreeing, asked to leave their e-mail-address in order to be contacted again. Data and email-addresses were stored separately from each other in order to guarantee anonymous responding. All participants gave their informed consent prior to the online poll. The study complies with the Declaration of Helsinki and is in accordance with the ethical standards of the institutional-and/or national research commitment.

### Measurements

#### Environmental- and social circumstances regarding SARS-CoV-2

We assessed demographic information regarding subjects’ age and occupation, their living- and family situation, corona-related fears- and worries (current and those about the future). Furthermore, social behavior regarding new rules in times of the pandemic and a new way of thinking- and feeling since the virus’ massive spreading was assessed.

#### Behavioral items regarding Corona (BCI)

As we came up with the questions regarding the SARS-CoV-2 pandemic ourselves, we calculated reliabilities for those items, in order to proof internal consistency. Furthermore, we calculated a factor analysis in order to be able to cluster items into higher order factors and to be able to associate those higher order factors with SMU as well as with factors that we expected to be protective from mental disorders, such as resilience, and coping strategies.

Therefore, a principal component analysis (PCA) with VARIMAX rotation was calculated (Eigenvalue > 1, maximum iteration: 25, loading > .4), containing 21 items that asked about behavior during the outbreak of SARS-CoV-2 in Germany as well as during the lockdown. Kaiser, Meyer and Olkin (KMO)—value was .785, and, therefore, acceptable in order to calculate the PCA. Four factors were extracted: 1) Social Distancing (not meeting many people, keeping distance, etc.), 2) Hoarding, 3) Global future anxiety (anxiety about the nation’s future and global future), and 4) Hygienic measures (washing hands more often with soap-and disinfectant, rumination about germs).

#### Mental health

Assuming which psychiatric symptoms might currently be most prevalent, we decided to focus on DEP, HA, GAD, panic attacks and PAD, as well as OCD. Regarding DEP, we used the German Version of the Beck-Depression-Inventory [23, 24]. Cut-off for a clinical depression measured with the BDI-II was divided into “any DEP” (BDI-score starting from 13) and another variable, indicating “severe/major DEP” with a BDI-II-score higher than 28. PAD and GAD were assessed with the German version of the Primary- Health-Questionnaire [25, 26], HA with the German version of the Short Health Anxiety Inventory [27, 28], and obsessive-compulsive symptoms with the German version of the Obsessive-Compulsive Inventory-Revised [29, 30].

For OCD, the cut-off was set to 21 in accordance with Foa and colleagues [29]. Concerning HA, subjects above one SD of the current samples’ mean, were classified as highly health-anxious, as no clear cut-off is given in scientific literature [28, 31].

#### Protective factors

As we were, furthermore, interested in which way coping styles, resilience, and well-being might be associated with mental health outcome during stressful circumstances, we assessed those as well using the German version of the brief resilience scale [32, 33], and of the WHO-5 well-being index [34]. Coping styles were recorded with the brief COPE [35, 36]. It assesses four main coping styles, while we focused on “Positive Focus” in the current study. Furthermore, personality dimensions were measured [37], however, results will not be reported here. Instructions of scales (except resilience and personality) were adapted to “during the past two weeks” in order to ensure a focus on the current SARS-CoV-2 situation in Germany.

#### Statistical analysis

Descriptive statistics were analyzed regarding distribution of age, gender, life situation, occupation, social media use, corona exposition and worries regarding consequences of the Covid-19 pandemic.

Variables for psychiatric disorders were transferred into dichotomous variables in dependence of absence or presence of the respective disease, and frequencies assessed. In order to assess Resilience, and Coping Styles in association, variables were median-splitted and Odd’s Ratios (ORs) were calculated.

In case of multiple testing, Bonferroni correction for multiple comparisons was applied. Significant results are reported only if they survived this correction. For data analysis, SPSS Statistics 26 (IBM Corp., Somer, N.Y., USA) was used.

ANOVAs were calculated in order to detect differences of BCI in people with- and without mental disorders.

## Results

### Descriptive statistics

N = 949 subjects participated in the study. They were on average 28.9 years old (SD± 10.8), 79.5% (n = 754) female, 19.9% (n = 189) male, and 0.6% (n = 6) non-binary. More detailed descriptive results are presented in Table 1.

**Table 1:** Total numbers (N) and frequencies (percentage) of various demographic variables in the underlying sample of N = 949 participants.

	No.	%
**Gender**		
Female	754	79.5
Male	189	19.9
Non-binary	6	0.6
**Occupational status**		
Student	596	62.8
Office worker	251	26.4
Officials	25	2.6
Self-Employed	12	1.3
Other	46	4.8
Unemployed	9	0.9
**Relationship status**		
Single	349	36.8
Married	147	15.5
Relationship	428	45.1
Other	25	2.7
**Living Situation**		
Alone	148	15.6
Shared flat	178	18.8
With spouse/partner	266	28.0
With partner and children	89	9.4
With partner, children elsewhere	8	0.8
Without partner, with children	13	1.4
With parents/grandparents	247	26.0
**Corona-related variables**		
Currently in quarantine	31	3.3
Diagnosed with Covid-19	3	0.3
With Covid-19 diagnosed family member	56	5.9
Caring for someone with Covid-19	269	28.3

### Prevalence of mental disorders

Overall, prevalence of suffering from any of the measured mental disorders was 50.6% (N = 480). Therefore, half of the sample’s subjects were suffering from at least one disease. Regarding specific mental disorders, 35.3% expressed clinically depressive symptoms (BDI-Score > 13), 12.0% met the criteria of GAD, 5.4% those of a PAD. 21.4% experienced clinically obsessive-compulsive symptoms (OCI-Score >21) and 17.4% had above average HA-scores and can, therefore, be classified being highly health-anxious in the direction to somatoform disorder. Data regarding 12-month-prevalence of psychiatric diseases in the German population before this pandemic [10] report the following: 7.7% depression rate, 2.2% for GAD, 2.0% for PAD, 3.6% for OCD, and 3.5% for somatoform disorders. Numbers in our sample are much higher, and might already show trends towards public’s future problems after this emergency situation is over. No significant differences between genders were evident in this sample. Results are depicted in Table 2. More detailed tables of sociodemographic variables and prevalence of mental disorders are provided in the S1 File.

**Table 2:** 12-month-prevalence of various mental disorders in Germany before the Covid-19 pandemic, and prevalence assessed during the lockdown in total numbers (No.) and frequencies (%).

	12-month-prevalence Germany [10], N = 4484		Observed Prevalence current sample, N = 949		
	%	95% CI	No.	%	95% CI
*Any Mental Disorder*	27.7	[26.3–29.2]	480	50.6	[47.4–53.7]
*Any Depression*	7.7	[6.9–8.6]	335	35.3	[32.1–38.4]
*General Anxiety Disorder*	2.2	[1.8–2.8]	114	12.0	[9.9–14.1]
*Panic Disorder*	2.0	[1.6–2.5]	51	5.4	[4.0–6.8]
*Obsessive Compulsive Disorder*	3.6	[3.1–4.4]	203	21.4	[18.8–24.0]
*Somatoform Disorder (Health anxiety)*	3.5	[2.9–4.1]	165	17.4	[15.0–19.8]

Annotations: CI = Confidence Interval

### Behavior during the lockdown and mental disorders

Analyzes of variance regarding measured mental disorders revealed that subjects with PAD differed significantly in their Social distancing behavior, *F*_(1, 947)_ = 10.24, *p* = .001, as did subjects with GAD, *F*_(1, 947)_ = 14.95, *p* < .001. Subjects with high HA scored higher on Social Distancing, *F*_(1, 947)_ = 29.87, *p* < .001, as well as Hoarding, *F*_(1, 947)_ = 16.33, *p* < .001. Differences in subjects with OCD showed a trend toward Social Distancing, which did, however, not withstand correction for multiple comparisons, *F*_(1, 947)_ = 6.40, *p* = .012. Depressive subjects did not differ from non-depressive ones regarding lockdown-related behavior (all *p* > .10). Hygienic measures were not associated with any mental disorder.

### Protective factors

**Fig 1:**
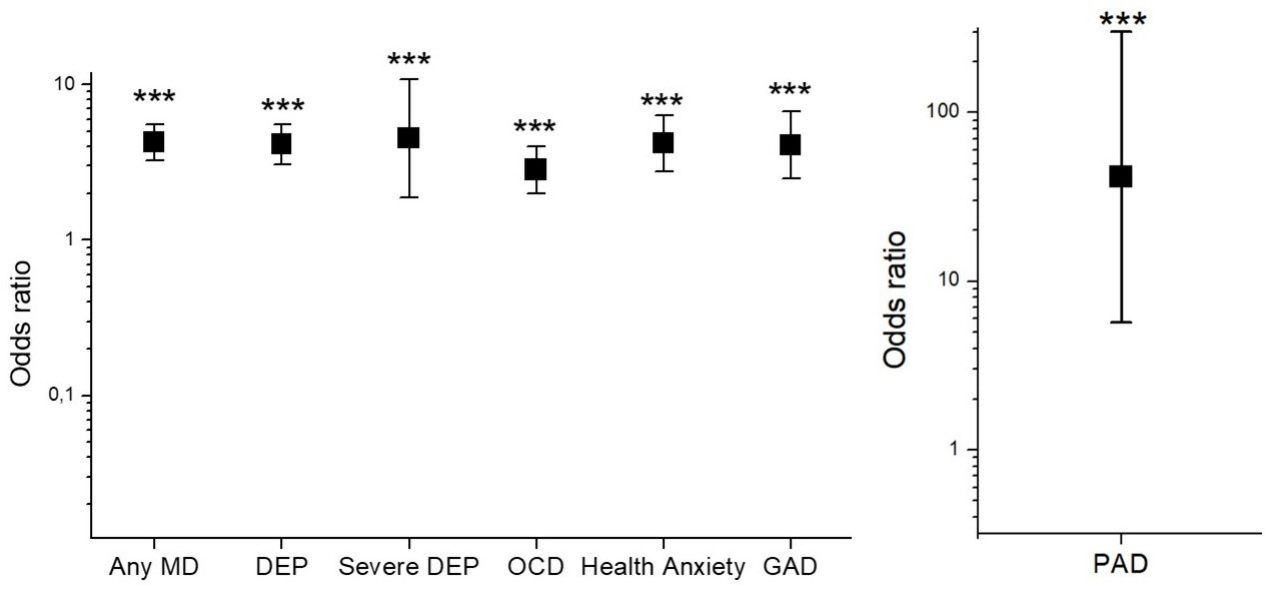
Odds ratios (log-scaled) of different mental disorders in association with resilience during Covid-19 pandemic in Germany. Any MD = Any mental disorder, HA = Health anxiety, PAD = Panic Disorder, GAD = General Anxiety Disorder, OCD = Obsessive Compulsive Disorder, DEP = Depression, Sev DEP = severe Depression. **p < .01, ***p < .001.

Regarding Resilience, analysis revealed higher Resilience in men compared to women, *t* = 4.83, *p* < .001, and a small positive correlation between age and Resilience, *r* = .09, *p* = .004. More resilient subjects had lower risks for all tested mental diseases: ORs for depression were lower in resilient subjects (OR = 4.12, *p* < .0001, 95% CI = 3.49–5.57), also for severe(major) depression (OR = 4.49, *p* < .0001, 95% CI = 1.86–10.80), PAD (OR = 41.44, *p* = .0002, 95% CI = 5.7–301.34), OCD (OR = 2.82, *p* < .0001, 95% CI = 1.98–4.00), HA (OR = 4.18, *p* < .0001, 95% CI = 2.75–6.73), as well as for GAD (OR = 4.07, *p* < .0001, 95% CI = 2.47–6.73). OR for any mental disorder in association with Resilience was 4.23, *p* < .0001, 95% CI = 3.21–5.57). Results are shown in Fig 1 below.

**Fig 2:**
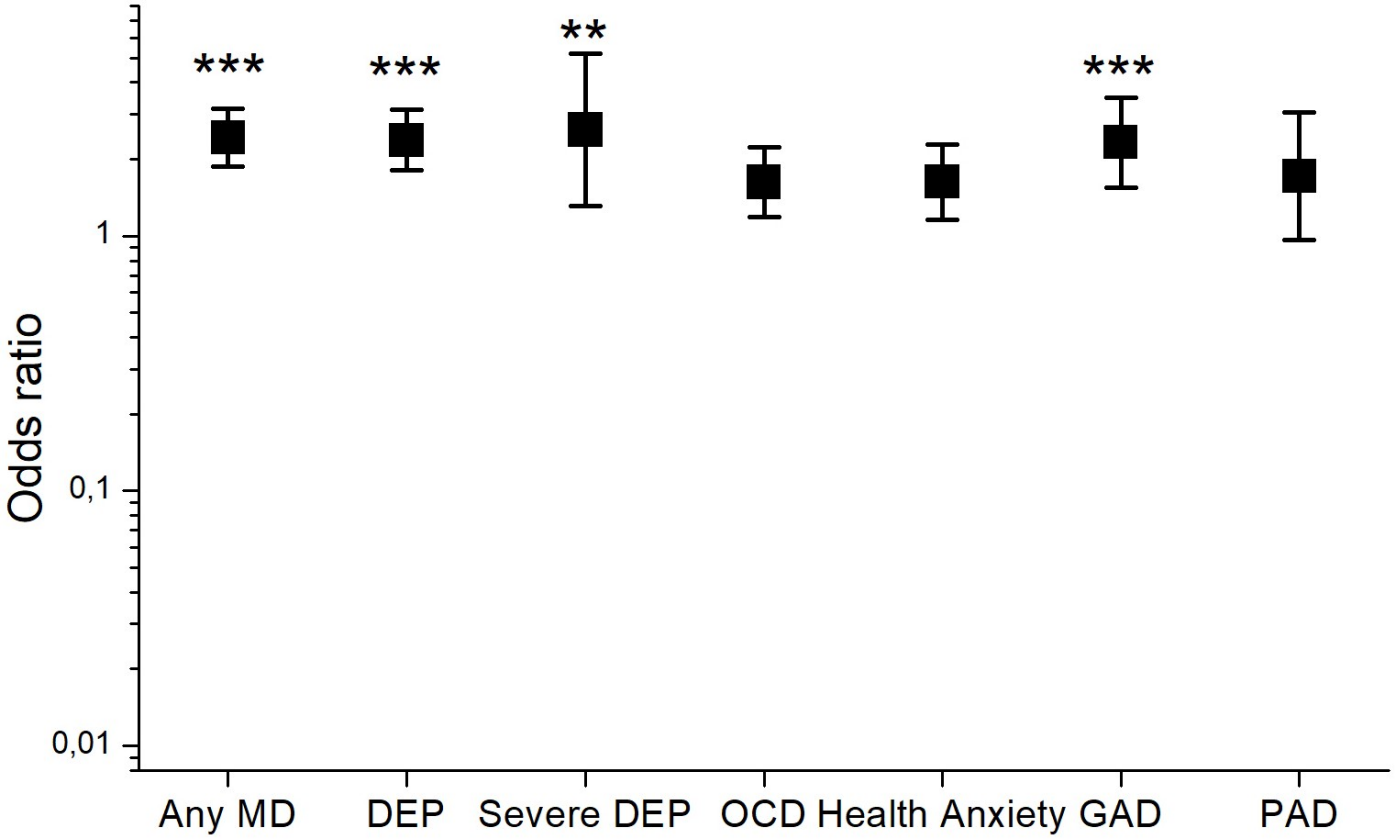
Odds ratios (log-scaled) of different mental disorders in association with coping style “Positive Focus” (PF) during Covid-19 pandemic in Germany. Any MD = Any mental disorder, HA = Health anxiety, PAD = Panic Disorder, GAD = General Anxiety Disorder, OCD = Obsessive Compulsive Disorder, DEP = Depression, Sev DEP = severe Depression. **p < .01, ***p < .001.

Regarding Coping Styles, Positive Focus was associated with lower risks for depression, (OR = 2.37, *p* < .0001, 95% CI = 1.80–3.12), severe(major) depression (OR = 2.61, *p* = .0063, 95% CI = 1.31–5.91), GAD (OR = 2.32, *p* < .0001, 95% CI = 1.54–3.49), and also with lower risks for any MD (OR = 2.43, *p* < .0001, 95% CI = 1.87–3.16). ORs were neither significantly different towards PAD (OR = 1.71, *p* = .066, 95%CI = .96–3.05), nor HA (OR = 1.63, *p =* .0048, 95% CI = 1.16–2.28), nor OCD (1.62, *p* = .0024, 95% CI = 1.18–2.22). Results are illustrated in Fig 2 below.

## Discussion

This study assessed the latest prevalence of various mental disorders during Covid-19 outbreak in Germany, and examined possible variables that could serve as protection against severe mental health problems, such as resilience and coping in N = 949 people:

Prevalence of depression, obsessive-compulsive disorder, panic- and general anxiety disorder were significantly higher than general national data reported on prevalence before the Covid-19 pandemic [10], indicating that the ongoing situation puts people under severe psychological stress: This needs to be monitored and demands immediate support to be established. Results are in line with data on prevalence regarding other major life threatening events, such as the Ebola outbreak, the Tsunami, or 9/11 [38–41] as well as with most recent data on mental health problems in China during the Covid-19 outbreak [5]. Especially prevalence of depression, with more than a third (35.3%) of the sample suffering from it, as well as GAD, PAD and OCD are alarming and already portrait a picture of which problems are going to occur after the lockdown is over, or if it is continuing for a longer time. This study presents a very early picture of general mental health during the first weeks of the pandemic situation in Europe–at least of those who completed our survey. Therefore, these data are also very important for future prognoses and could be relevant for considerations regarding how long the lockdown should go on or at what time maybe other options/solutions should be taken under consideration. As all the considerations are matters of “pros and cons”, mental health and well-being of a whole state should also be monitored and not to be left aside. If this situation should go on for a longer time, for example App-based therapeutic interventions could be helpful, as well as monitoring of mental health, in order to be able to intervene if symptoms deteriorate.

Our study was conducted at a relatively early phase of the lock-down in Germany (starting one week after the measurements were being effective, on March 27^th^ 2020). Specific government policies, e.g. compulsory mask wearing in public areas and stores, were introduced and obligatory around six weeks after the survey ended. Therefore, effects of mandatory mask wearing on studied mental problems cannot be reported here.

Therefore, future work should evaluate long-term mental health consequences of different government policies by comparing results from different countries and assessing associations with e.g. timing of specific legal requirements, risk communication, duration of lock-down and possible mental health interventions. We did, however, observe associations between mental health and corona-related behaviors such as social-distancing and hoarding. As Garbe and colleagues [42] reported associations of personality and stockpiling as well, these factors should be considered by policy makers—as they could be crucial for controlling the pandemic [43]. For example, Rieger [44] determined judgement anxiety as a factor that is related to compliance to wear masks in Germany and gave important recommendations how to address specific target groups in order to increase mask wearing and compliance. Also, in times of protective equipment shortage, thoughtful risk communication is required to ensure supply for healthcare workers and prevent people from hoarding [45]. Furthermore, psychological aspects like mental health problems, as well as discrimination and stigma which might arise due to infection or quarantine, should directly be addressed by politicians via broadcasts, social media and other online services in order to prevent more social isolation and increase of mental health problems [46, 43].

Furthermore, it is crucial to study factors, which are possibly protective against mental disorders, in order to be able to inform the population about possible ways to cope with the situation and to suffer less. We found out that 1) resilient subjects were less likely to suffer from any mental disorder, and 2) that focusing on positive situational aspects as coping style was associated with lower risks for depression, GAD, and HA. Especially the risk for panic disorder was substantially higher in low resilient subjects. Training of resilience in subjects prone to panic attacks could potentially prevent those from developing a PAD. This could be a very powerful starting point in dealing with impact of Covid-19 on mental health:

There are several online interventions that focus on improving resilience and coping styles [47, 48], also designed at times of facing traumatic events [49]. Such interventions could be very helpful for the current situation, as they can be done online-, and, therefore, made available to a great audience. These could prevent further deterioration or development of mental disorders [47]. Interventions could also be adapted to groups being particularly affected by the pandemic such as healthcare workers [50], or chronically ill, as it was done e.g. during/after SARS [51].

### Limitations of the study

This study assessed 949 subjects in Germany within one week (March 27th-April 3rd 2020). Although this is a comparatively high number, several limitations have to be addressed: Mean age of our sample was 28.97 years, with 62.8% students, and in total a sample with higher education (43.1% with university degree), hence, also with a higher socio-economic status (SES). Whereas this reduces the representativeness of the sample, it can also be assumed that prevalence in the broader population is even higher, as SES is a mediator for physiological and mental health [52] with lower prevalence alongside higher SES [10]. Whereas Jacobi and colleagues [10] used a structured clinical interview to assess prevalence, online-questionnaires were used here. Therefore, our prevalence-estimations might not be as accurate as those [10], although applied questionnaires are validated and clinically broadly used. As the purpose of the study was to catch a glance of what is happening to the population during this pandemic on a mental health level, online questionnaires where the only realizable solution. Nonetheless, our data are congruent with latest prevalence rates during the Covid-19 outbreak in China [5]. While the use of questionnaires specifically designed for COVID-19 would have been preferable, such measurements had not been published by the time our study was conducted (March 27th and April 3rd, 2020). Fortunately, new measurements have been developed and validated [7, 53–55].

Moreover, observed effects do not reveal causal relationships, as this is a cross-sectional study and we do not directly have a baseline of mental health issues before the survey. Nevertheless, participants are going to be observed longitudinally, and, therefore, it will be possible to make more clear inferences on causal relationships later on.

## Conclusion

To our knowledge, this is the first study in Germany portraying and revealing the severe impact of Covid-19 on the society—not only as a direct physiological, but also as a psychological burden. It is, therefore, crucial to monitor the development of reported prevalence and to find possible treatment solutions for a country (or a world) after the pandemic is “under control”. Furthermore, economic consequences of the pandemic might deteriorate the psychiatric states even further. It is not only substantial to have enough ICU-equipment, it is also crucial to care for the traumatized people after the outbreak, and to provide the necessary infrastructure. In order to observe the development of addressed mental health issues, longitudinal observation/assessment of the participants will show if symptoms deteriorated or improved.

## Supporting information

S1 File (DOCX)

S1 Data (SAV)
