# Supplementary material for: Covid-19—Beyond virology: Potentials for maintaining mental health during lockdown
Source: PLoS One. 2020 Aug 4;15(8):e0236688. doi: 10.1371/journal.pone.0236688 (PMC7402475; doi:10.1371/journal.pone.0236688)
Supplement: S1 File — (DOCX) [file pone.0236688.s001.docx]

Supplementary material: Prevalence of assessed psychiatric diseases

**Table 1.**

Prevalence of depression and socio-demographic variables

| **Variables** | **Prevalence (95%CI)** | **No.** 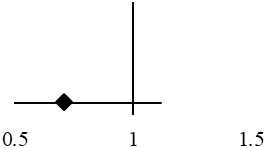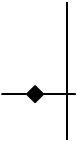 |  | **Odds Ratio (OR; 95%CI)** |
| --- | --- | --- | --- | --- |
| **Overall** | 35.3 (32.2-38.5) | 335/949 |  | - |
| **Gender** |  |  |  |  |
| Female | 35.5 (32.2-38.9) | 286/754 |  | 1 (ref) |
| Male | 33.3 (27.0-40.2) | 63/189 |  | 0.88 (0.75-1.03) |
| **Age(years)** |  |  |  |  |
| 18-40 | 36.2 (32.9-39.4) | 299/827 |  | 1 (ref) |
| 40+ | 29.5(22.1-37.7) | 36/122 |  | 0.74 (0.49-1.12) |

*Annotations: CI = Confidence interval*

**Table 2.**

Prevalence of severe depression and socio-demographic variables

| **Variables** | **Prevalence (95%CI)** | **No.** 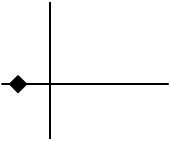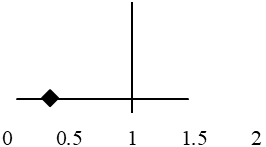 |  | **Odds Ratio (OR; 95%CI)** |
| --- | --- | --- | --- | --- |
| **Overall** | 4.2(2.9-5.6) | 40/949 |  | - |
| **Gender** |  |  |  |  |
| Female | 4.4(2.9-6.0) | 33/754 |  | 1 (ref) |
| Male | 2.6 (0.5-5.3) | 5/189 |  | 0.76 (0.63-0.91) |
| **Age(years)** |  |  |  |  |
| 18-40 | 4.6 (3.3-6.1) | 38/827 |  | 1 (ref) |
| 40+ | 1.6 (0.0-4.1) | 2/122 |  | 0.35 (0.08-1.45) |

*Annotations: CI = Confidence interval*

**Table 3.**

Prevalence of obsessive-compulsive disorder and socio-demographic variables

| **Variables** | **Prevalence (95%CI)** | **No.** 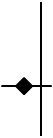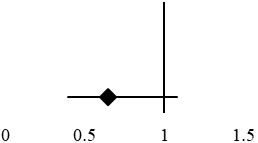 |  | **Odds Ratio (OR; 95%CI)** |
| --- | --- | --- | --- | --- |
| **Overall** | 21.4 (18.8-24.1) | 203/949 |  | - |
| **Gender** |  |  |  |  |
| Female | 21.0 (17.9-24.0) | 158/754 |  | 1 (ref) |
| Male | 22.2 (16.4-28.0) | 42/189 |  | 0.90 (0.77-1.06) |
| **Age(years)** |  |  |  |  |
| 18-40 | 22.2 (19.4-25.0) | 184/827 |  | 1 (ref) |
| 40+ | 15.6 (9.0-22.1) | 19/122 |  | 0.65 (0.39-1.08) |

*Annotations: CI = Confidence interval*

**Table 4.**

Prevalence of panic disorder and socio-demographic variables

| **Variables** | **Prevalence (95%CI)** | **No.** 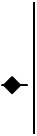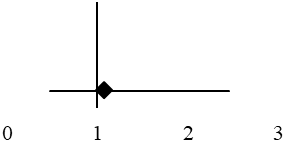 |  | **Odds Ratio (OR; 95%CI)** |
| --- | --- | --- | --- | --- |
| **Overall** | 5.4 (3.9-6.8) | 51/949 |  | - |
| **Gender** |  |  |  |  |
| Female | 5.7 (4.1-7.5) | 43/754 |  | 1 (ref) |
| Male | 3.2 (1.0-5.9) | 6/189 |  | 0.77 (0.65-0.92) |
| **Age(years)** |  |  |  |  |
| 18-40 | 5.3 (3.9-6.8) | 44/827 |  | 1 (ref) |
| 40+ | 5.7 (1.6-10.7) | 7/122 |  | 1.08 (0.48-2.46) |

*Annotations: CI = Confidence interval*

**Table 5.**

Prevalence of generalized anxiety disorder and socio-demographic variables

| **Variables** | **Prevalence (95%CI)** | **No.** 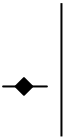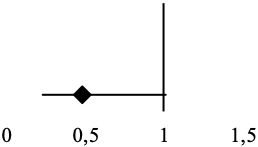 |  | **Odds Ratio (OR; 95%CI)** |
| --- | --- | --- | --- | --- |
| **Overall** | 12.0 (10.0-14.1) | 114/949 |  | - |
| **Gender** |  |  |  |  |
| Female | 12.7 (10.3-15.2) | 96/754 |  | 1 (ref) |
| Male | 7.9 (4.3-12.1) | 15/189 |  | 0.78 (0.66-0.92) |
| **Age(years)** |  |  |  |  |
| 18-40 | 12.8 (10.6-15.0) | 106/827 |  | 1 (ref) |
| 40+ | 6.6 (2.5-11.5) | 8/122 |  | 0.48 (0.23-1.01) |

*Annotations: CI = Confidence interval*

**Table 6.**

Prevalence of somatoform disorder/health anxiety and socio-demographic variables

| **Variables** | **Prevalence (95%CI)** | **No.** | 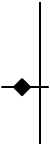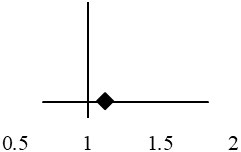 | **Odds Ratio (OR; 95%CI)** |
| --- | --- | --- | --- | --- |
| **Overall** | 17.4 (14.9-19.9) | 165/949 |  | - |
| **Gender** |  |  |  |  |
| Female | 17.9 (15.1-20.5) | 135/754 |  | 1 (ref) |
| Male | 14.8 (10.1-20.1) | 28/189 |  | 0.89 (0.76-1.05) |
| **Age(years)** |  |  |  |  |
| 18-40 | 17.2 (14.6-20.0) | 142/827 |  | 1 (ref) |
| 40+ | 18.9 (12.3-26.2) | 23/122 |  | 1.12 (0.69-1.83) |

*Annotations: CI = Confidence interval*

**Table 7.**

Prevalence of any psychiatric disorder and socio-demographic variables

| **Variables** | **Prevalence (95%CI)** | **No.** 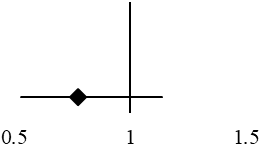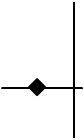 |  | **Odds Ratio (OR; 95%CI)** |
| --- | --- | --- | --- | --- |
| **Overall** | 50.6 (47.4-53.5) | 480/949 |  | - |
| **Gender** |  |  |  |  |
| Female | 50.9 (47.2-54.7) | 384/754 |  | 1 (ref) |
| Male | 48.1 (41.3-55.6) | 91/189 |  | 0.86 (0.72-1.03) |
| **Age(years)** |  |  |  |  |
| 18-40 | 51.4 (47.7-54.7) | 425/827 |  | 1 (ref) |
| 40+ | 45.1 (36.1-54.1) | 55/122 |  | 0.78 (0.53-1.14) |

*Annotations: CI = Confidence interval*
